# Evidence-Based Genetic Education of Non-Genetic-Expert Physicians: Experiences Over Three Decades in Amsterdam

**DOI:** 10.3389/fgene.2019.00712

**Published:** 2019-08-02

**Authors:** Martina C. Cornel

**Affiliations:** Amsterdam UMC, Vrije Universiteit Amsterdam, Clinical Genetics, Section Community Genetics, Amsterdam Public Health Research Institute, Amsterdam, Netherlands

**Keywords:** knowledge, genetics, genetic education, eLearning, competences, curriculum, continuing professional development

## Abstract

To study and improve the competences of health-care workers in the domain of genetics, attention needs to be paid to attitudes, activities, knowledge, and changes in performance. Three decades of research on genetic education for non-genetic-experts in Amsterdam are summarized, including both local and international collaborative efforts. Evidence shows that assessment of learners’ needs and the definition of competences have driven slow but gradual improvement in genetics competence among non-geneticists. Attitudes and behavior are mainly influenced by face-to-face training. eLearning modules can serve to increase knowledge in a large number of participants in a rapidly changing field. Materials developed for accredited courses will sometimes be used for reference or just in time learning. Taking a theoretically informed evaluation approach, it has been possible to demonstrate satisfaction, improved knowledge, and self-reported behavioral change, although measuring effects on health-care practice and population health remains challenging. A flexible approach is needed to serve learners’ needs in a field with many upcoming challenges.

## Introduction

In the last decades, genetics and genomics research has generated many new insights, but the implications for health care so far have been modest. The publication of the sequence of the human genome was seen as a potential turning point. On 26 June 2000, Francis Collins stood next to the President of the United States, who announced the publication of the first survey of the entire human genome, and stated that “It will revolutionize the diagnosis, prevention, and treatment of most, if not all, human diseases” (Collins, 2010). However, 10 years later, Francis Collins concluded that while the revolution had not yet arrived, a few powerful new drugs against cancer and predictive genetic tests for a dozen conditions had become available (Collins, 2010). To enable the revolution, education of health-care providers was presented as one of the factors needed. This education should increase genetic knowledge and skills in physicians in domains outside of clinical genetics. In the last few decades, several studies have been performed in the Community Genetics Research group in Amsterdam, the Netherlands, in collaboration with colleagues from other countries, to improve genetic knowledge and skills and evaluate the approaches used. The tradition in Amsterdam is characterized by a strong evidence-based approach. In this paper, I will review lessons learned from three decades of genetic educational research. In some studies, there was a focus on activities, attitudes, and knowledge, as defined by experts, but there was also attention on educational needs as defined by nonexperts. Evaluation took place on different levels, including satisfaction, increase of knowledge, behavioral change, and use in health-care practice.

## Activities and Attitudes

In the Netherlands, general practitioners (GPs) have a role as gatekeepers in health care. If a couple has questions about their risk of having a child with congenital anomalies, they will first go to their GP, who can refer them to a specialist such as a clinical geneticist or obstetrician. A PhD thesis published in 1997, reported how well GPs in 1989 performed their task with regard to identifying and informing couples who are at increased risk of having a child with a congenital disorder (De Smit, 1997; Baars et al., 2003). A random sample of 124 GPs from the province of Noord-Holland (including Amsterdam) received a questionnaire, and 74% responded. Genetic counseling was defined as the provision of information on the chances of hereditary diseases and congenital disorders and on the possibilities of genetic examination, prenatal diagnosis, and pregnancy termination. Ten years later, the same GPs were investigated again, and 72% responded (Baars et al., 2003). The GPs recorded information on potential risk factors in their database for “previous child with congenital anomaly” in 57% (in 1989) and 63% (in 1999) and information on a serious congenital disorder in the close family in 15 and 13%, respectively. Information on consanguinity was recorded in 19 and 23%, respectively. GPs often gave oral information and, rarely, written information. In 1989, 82% supported directive counseling, and in 1999, this percentage had increased to 87% [measured as (strong) support for the statement “Genetic counseling should push the decisions of women and their partners on carrying out prenatal diagnosis in the right direction”]. The stance of clinical geneticists was that genetic counseling should be nondirective, especially for reproductive decisions, given the preference sensitive and value-laden decisions. The percentage of GPs that reported that they “(almost) always” referred women to a clinical geneticist for genetic counseling increased from 20% to 37%. The authors concluded that there was limited improvement in the GPs’ activities over the 10-year time span (Baars et al., 2003). Around that time, an epidemiological study in the Northern Netherlands showed that 17% of couples who had a child with a congenital disorder were referred for genetic counseling (Cornel et al., 1992), and 10 years later, this percentage was 18% (Sikkens et al., 2002). The authors concluded that despite the increasing familiarity with genetics, the uptake of genetic counseling had not increased.

### Curricula

Given the slow improvement of activities and attitudes, one might wonder what was taught in medical schools and specialist training. Given the fast developments in genetics, internationally, it was felt that more insight was needed in the content of medical education, and the “Genetic Education for Nongenetic Health Professionals” project (GenEd) was performed in 11 European countries (Challen et al., 2005). Wide variation was reported, and many countries lacked explicit genetics in their undergraduate, postgraduate, and continuing education. As part of the GenEd project, the medical curricula in eight medical faculties in the Netherlands in the year 2002 were investigated, as well as genetics in postgraduate training for non-genetics health-care professionals (Plass et al., 2006). Written documentation was studied and checked for accuracy with the genetic educators from each medical school. All medical curricula in The Netherlands used a list of “final goals of basic medical training.” Two of the 328 health issues were genetic: “request for genetic evaluation” and “suspicion of genetic/congenital anomaly,” and three were frequently used in the context of genetics: “increased risk (positive test result of screening),” “request for preventive evaluation,” and “request for information.” Health issues were formulated in a rather general way, making it hard to identify specific fields of medicine. Genetics was relatively invisible in the curricula, often being integrated within a course (e.g. reproduction, developmental disorders). Thus, Plass et al. reported that it was hard to estimate the time spent on genetics.

As a very general development, many medical faculties in the Netherlands around 2002 used “problem-based” and increasingly “competence-based” curricula. Competences became the formal backbone of medical education in the Canadian Medical Education Directions for Specialists (CanMEDS) framework (Frank and Langer, 2003), which was used increasingly in medical faculties for graduate and postgraduate trainings. Thus, similar results might have been found for other basic sciences or fields of applied medicine (e.g., anatomy, histology, pathology, rehabilitation). Plass et al. (2006) included postgraduate training in their analysis. Out of 27 medical specialist training programs, only three (other than clinical genetics) indicated formal genetics training (obstetrics and gynecology, neurology and paediatrics) (Plass et al., 2006). The training of MDs for intellectually disabled people, which was not a recognized medical specialism at that time, also formally included genetics. As for continued education, MDs were obliged to follow postgraduate training, but they were free to choose from many topics. Only a few genetics courses were available. A postgraduate genetics course for obstetricians/gynecologists existed, and a genetics course for cardiologists was being developed. The authors expressed the concern that genetics education was not only invisible but also insufficient.

## Knowledge

Before the year 2000, clinical genetics had a strong focus on children with congenital anomalies. The population of patients referred was mainly children and their parents: couples looking for a diagnosis for their child and often considering reproductive decisions. Couples with a relative with a congenital anomaly were also referred for reproductive planning. After 2000, oncogenetics became a more frequent reason for referral. Medical curricula had not changed very much, and genetic issues were scarcely mentioned in the official final training goals. A study was done to evaluate knowledge of genetics relevant for daily practice in students nearing graduation (Baars et al., 2005b). Out of 855 questions on genetics selected from medical examinations and literature, 215 questions were selected for an examination administered by computer. These 215 questions were assessed by clinical geneticists for their relevance to daily medical (non-genetic) practice and classified as “essential,” “desirable,” and “too specialized.” Participants were students in the final years of clerkships in seven out of eight of the medical faculties in the Netherlands. None of the students scored over 95% for “essential” knowledge, approximately a quarter of the students scored 60% or more for “desirable” knowledge, and most of the students scored over 40% for “too specialized” knowledge. Of the participants, 93% failed according to the cutoff score as defined by non-genetic health-care providers. Apparently, their knowledge was relatively good for issues that were less relevant for daily practice, while “essential” knowledge was often insufficient. It was hypothesized that in genetic education, too much attention is paid to specialized topics. The advice was that time spent on genetics should be spent more efficiently and should focus on knowledge that is relevant for daily practice (Baars et al., 2005b). While much of the research in Amsterdam focused on medical students and primary care physicians, the studies on knowledge also included gynecologists and pediatricians (Baars et al., 2005a). Average scores increased from GPs to gynecologists, pediatricians, and the clinical geneticist validation group. Overall genetic knowledge showed deficiencies for non-geneticist health-care providers. There was a specific lack of knowledge about DNA testing (Baars et al., 2005a).

## Competences Needed

To develop curricula for medical faculties, it is essential to define what a health-care professional needs to know and which competences are needed. A group of relevant health professionals and patients developed a set of core competences for different groups of health-care providers: GPs; genetic nurses/midwives; medical specialists in fields other than genetics; specialist nurses, specialist midwives, and specialist allied health professionals; specialist dentists; clinical geneticists; genetic specialist nurses or genetic counselors; molecular geneticists; cytogeneticists; and biochemists/biomedical scientists (Skirton et al., 2010). This was done in a collaborative project funded by the European Union: EuroGentest and under the auspices of the European Society of Human Genetics Education Committee. An exhaustive process of consultation took place, both with relevant health professionals and patient groups.

General competences include: to recognize individuals who may have a genetic condition; to be able to discuss this with patients and to refer; and to manage patients with a genetic condition and coordinate the care with other health-care workers. More specific competences were defined for clinical geneticists, genetic nurses or genetic counselors, molecular geneticists, cytogeneticists, and biochemists. These sets of competences can help countries to adjust their education and genetic service delivery systems for the future, according to a coherent set of standards (Skirton et al., 2010).

## Developing Educational Modules Based on Needs

Building on the core competences defined by Skirton et al. (2010) and starting with assessment of the needs of primary health-care professionals, a comprehensive educational program for genetics was developed (Houwink et al., 2015). Given the fast developments in genetics, a flexible approach was chosen, which would also be suitable for future challenges in other fields of genetics. Midwives and GPs first reported their needs in a focus group study (Houwink 2011), after which prioritization took place in a Delphi procedure (Houwink et al., 2012). The top three genetic competencies were “recognizing signals that can indicate a hereditary component of a disease,” “evaluating indications for referral to a clinical genetics centre,” and “knowledge of the possibilities and limitations of genetic tests” (Houwink et al., 2012). These general competencies could in theory be applied in different fields (e.g. reproduction, cardiogenetics, oncogenetics). As the focal theme of the Dutch College of General Practitioners (NHG) was oncology, the competences were elaborated for oncogenetics. Three products were developed: an online continuing professional development module on oncogenetics (G-eCPD), a live genetic CPD module (interactive program taking oncogenetics as a model condition), and a supportive website (www.huisartsengenetica.nl, “GP and genetics”). For the evaluation of learning outcomes, Kirkpatrick’s model was used (Kirkpatrick, 1967). The first level of Kirkpatrick’s involves satisfaction, the second level knowledge, the third level behavioral change, and the fourth and highest level organizational change and health gain.

The eCPD was evaluated in a randomized controlled trial in 80 GPs (Houwink et al., 2014). Satisfaction was high, knowledge increase showed moderate effect sizes, also at 6 months follow-up (Houwink et al., 2014). The evaluation of learning outcomes at the lower levels is relatively simple, but providing evidence of behavioral change, organizational change, and health gain are challenging. The difficulty is partly related to the follow-up needed. As for the website, visitor numbers and percentage returning visitors could be reported. Website visitors often looked for information on basic genetics (drawing family trees, family history taking), which was not expected initially (Houwink et al., 2015). Participants of the live training reported more frequent referral of patients to the clinical genetics centers (68%) vs. 29% of participants of the eCPD (Houwink et al., 2015). On a regional population level, however, referral did not increase in the year after the modules. This might be due to the small number of participants and small number of referrals as compared with that of the entire region. A longer follow-up time and modules on other topics (e.g. reproduction and development, cardiogenetics) might be needed to achieve significantly more referrals by GPs.

## eCPD and Website on Multiple Topics for Multiple Countries

Many European countries face similar challenges related to genetic education. A European Union postgraduate education project, Gen-Equip, led by Prof. Heather Skirton, developed online continuing professional development (CPD) modules on nine topics (Paneque et al., 2017). As the challenges for genetics in primary care in different countries are very similar, the joint efforts made it possible to develop similar materials in six European languages. The online modules are supported by a website and webinars. The materials are available for free. Knowledge and skills increased significantly, and self-reported behavior changed (Jackson et al., 2019). Just like in the Houwink study, not only increasing skills in collecting family information and drawing pedigrees were mentioned but also knowing how to explain genetics to patients. Behavioral change was evidenced by participants who organized genetic training for their colleagues. While the modules were accredited for continuous education, users frequently did not ask for a certificate but came back for the materials to use “just-in-time.”

## Current Situation in Netherlands

Clinical geneticists are involved in face-to-face training to groups of not only primary care physicians but also a diversity of other specialties and medical students. Online modules are developed for a range of rare diseases (e.g. monogenic subtypes of diabetes) and general issues (e.g. recognizing rare diseases), some of these in collaboration with patient organizations. Specialists other than clinical geneticists can now order some DNA tests (mainstreaming), and some specific modules for these purposes have been developed. A problem of this fragmented approach is that learners may not see certain challenges until they face them in practice. If they are unknowingly unable on, for instance, variants of unknown significance or the responsibilities toward family members, they may not request support until they are overwhelmed. While some of the funding for previous genetic education projects came from the National Genomics Initiative, currently, no specific large-scale funding is available. The limited availability of funding leads to fragmentation, where the evidence-based approach to education and evaluation may be more difficult to achieve on a long-term and/or national scale. Evaluation at the higher levels of Kirkpatrick’s requires a long-term involvement and may be difficult to achieve without dedicated funding.

## Conclusion

In the last decades, both genetic services and medical education underwent major changes. Problem-based learning and competence-based curricula gained importance, as did online learning modules. Given the underuse of the potential of genetics for health care, all of these strategies can help to improve the knowledge and skills relevant for daily practice. The challenge is to adapt to external changes in terms of technology and resources and patients’ and learners’ needs; particularly, learners are unknowingly unable for some aspects. Using an evidence-based approach to the development of modules can help to have most impact: learners’ needs can be served best, and the flexible approach can integrate the challenges of tomorrow.

## Author Contributions

MC wrote the article.

## Conflict of Interest Statement

The author declares that the research was conducted in the absence of any commercial or financial relationships that could be construed as a potential conflict of interest.
